# Fat-Free Mass and Bone Mineral Density of Young Soccer Players: Proposal of Equations Based on Anthropometric Variables

**DOI:** 10.3389/fpsyg.2019.00522

**Published:** 2019-03-29

**Authors:** Rossana Gomez-Campos, Thiago Santi-Maria, Miguel Arruda, Thiago Maldonado, Altamiro Albernaz, Marco Schiavo, Marco Cossio-Bolaños

**Affiliations:** ^1^Universidad Autónoma de Chile, Talca, Chile; ^2^Faculty of Physical Education, State University of Campinas, Campinas, Brazil; ^3^Sociedade Esportiva Palmeiras, São Paulo, Brazil; ^4^Department of Physical Activity Sciences, Faculty of Education Sciences, Catholic University of the Maule, Talca, Chile

**Keywords:** soccer, equations, bone mineral density, fat-free mass, young people

## Abstract

**Background:**

The assessment of body composition may assist in optimizing competitive efficiency and monitoring the success of training regimes for young soccer players. The purpose of this study was to determine the predictors for Fat-Free Mass (FFM) and Bone Mineral Density (BMD) of young soccer players. Also, the goal was to propose regression equations to estimate FFM and BDM through anthropometric variables.

**Methods:**

One hundred and sixty-seven young soccer players ages 10.0 to 19.9 years old were studied. Weight, height, trunk-cephalic length, right arm circumference, diameter of the humerus, and length of the foot were assessed. FFM and BDM were determined by using dual X-ray absorptiometry (DXA). Maturity status using Peak Height Velocity (PHV) was calculated.

**Results:**

Maturity status, weight, and circumference of the relaxed arm positively related to the FFM (*R*^2^ = 41–66%). Similarly, PHV, weight, diameter of the humerus, and length of the foot explained BDM in both groups of soccer players (goalkeepers and filed players) (*R*^2^ = 45–82%). Six equations to predict FFM (*R*^2^ = 62–69%) and six to predict BDM (*R*^2^ = 69–90%) were created. Chronological age had a limited use for predicting FFM and BDM.

**Conclusion:**

Results suggested the use and application of the regression equations as a non-invasive alternative for everyday use in soccer clubs.

## Introduction

The assessment of body composition can provide valuable information about the changes that arise during the stages of training (Wittich et al., 1998). On the other hand, monitoring bone health during physical growth is relevant because it allows identification of low accumulation of minerals or risk of osteoporosis in due to low bone mineral density (BMD) in youth (Zemel et al., 2011).

Therefore, The Mechanostat Theory (Rauch et al., 2004) can be used to explain the muscle-bone relationship where larger muscles exert greater traction on the connector bones. In this sense, bone strength can be affected by physical exercise in two ways: from high impact loads associated with exercise and through indirect osteogenic muscle development in a given area where greater stress is exerted on the bone (Rahnama et al., 2005; Vicente-Rodriguez, 2006).

Currently strong empirical evidence exists that demonstrates that physical exercise affects the normal development of muscles and bones (Daly, 2007; Krustrup et al., 2009; Helge et al., 2010). Thus, the evaluation of these parameters can help optimize competitive efficiency and monitor the success of the training regimes of young soccer players.

Essentially, the analyses of body composition and bone health are often carried through dual X-ray absorptiometry (DXA). This method is considered ideal for use in children due to its wide availability, fast scanning, low radiation exposure (Zemel et al., 2011). However, this methodology is considered costly and inaccessible to most soccer teams, especially for young athletes (Shim et al., 2014) belonging to sports clubs without laboratories and sophisticated equipment.

In this context, anthropometry emerges as an alternative that is applicable to everyday situations. In addition to being useful, it has numerous advantages, such as the ease of administration and low cost (Santi-Maria et al., 2015). Moreover, as far as it is known, no national and international studies have proposed regression equations based on anthropometric variables that allow estimating the Fat-Free Mass (FFM) and BMD of young soccer players. Adolescence is the best time to gain BDM as well as to modify the size of the skeleton and its structure in response to mechanical loads (Marcus, 2001).

The hypothesis for this study was the following: the arm and muscle circumferences may be strongly related to the FFM, and the length and diameter of the humerus bone may have a close relationship with BMD in young soccer players. These variables are routinely assessed in as part of the control for physical growth and body composition. Additionally, chronological age, maturity status, and body weight may be important predictors of the aforementioned variables.

Thus, this study had two objectives. First, (a) to determine the predictors of FFM and BMD of young soccer players. Second, (b) to propose regression equations to estimate FFM and BMD with anthropometric variables.

## Materials and Methods

### Subjects

A descriptive correlational study was designed to study the FFM and BMD of 167 young soccer players from the Sociedade Esportiva Palmeiras Sports Club, in São Paulo, Brazil. The young athletes belonged to the Centro de Formação de Atletas (Centre for Training Athletes). They were selected by convenience non-probabilistically, agreeing to participate voluntarily. The age range fluctuated between 10.0 and 19.9 years old. Game positions for this study included: Goalkeepers (*n* = 22), full-backs (*n* = 27), defenders (*n* = 22), defensive midfielders (*n* = 63), midfielders (*n* = 29), and strikers (*n* = 29). The sample was divided into two groups: goalkeepers and field players.

### Data Collection

Evaluations were carried out at the beginning of the competitive season. Study subjects aged less than 13.9 years trained 4 times a week for 60–90 min/day (a competition) and had 3 years of experience in the sport. Players aged between 14.0 and 16.9 years trained 5 times a week 90–120 min/day (a competition) and had 5 years of experience. Individuals aged over 17.0 years trained 6 times a week 90–120 min/day (a competition) and had 6 years of experience. All players of legal age signed the informed consent form. For players less than 17.9 years old, parents, and/or guardians signed the informed consent form. At the same time, the underage minors were asked to sign the consent form if they agreed to participate. All players signing the informed consent form and underage players between the ages of 10.0 to 19.9 years with parental or guardian consent were included in the study.

Players with out medical clearance on the day of the assessment were excluded. The study was conducted in accordance with the guidelines established by the Ethics Committee of the School of Medical Sciences of the University of Campinas (São Paulo, Brazil- #2015-088). Written informed consents were collected from all participants prior to their inclusion in the study.

Anthropometric evaluations and the DXA scan were carried out in the club facilities (laboratory) in February 2015 (Monday-Friday) from 8 a.m. to 10 a.m. Four trained professionals were in charge of the evaluation process (three in anthropometry and one in DXA). The professional in charge of the DXA evaluations had the necessary certification for using and operating the equipment.

Anthropometric variables were evaluated following the Protocol of the International Society for Advancement of Kinanthropometry [Society for Advancement of Kinanthropometry [ISAK], 2001]. Weight, height, sitting height, circumference, diameter of the elbow, and length of the foot were measured twice. The Technical Error of Measurement ranged from 2 to 3%. Reproducibility ranged from *r* = 0.95 to 0.98.

Body mass (kg) was evaluated with a balance (SECA, Hamburg) with precision of 0.1 kg. Height was measured with a stadiometer (SECA, Hamburg) with a precision of 0.1 cm. Sitting height was measured on a wooden bench (flat box 50 cm high). A stadiometer (SECA, Hamburg) with 0.1 cm precision was used. The circumference of the relaxed arm was evaluated on the right side of the body with a metal measuring tape of SECA brand and graduated in millimeters with a precision of 0.1 cm. The diameter of the humerus bone and length of the foot were evaluated with a Harpenden adipometer with a range of 0 to 2.00 m and with a precision of 1 mm. Both variables were measured on the right side of the body.

Maturity status was determined by the Peak Height Velocity (PHV) obtained from a regression equation proposed by Mirwald et al. (2002). This method included the standing height, sitting height, length of the legs (standing height – sitting height), decimal age, and its interactions. Maturity status was constructed using 1 year intervals, represented as -4, -3, -2, -1, 0, 1, 2, 3, and 4 PHV.

Fat-Free Mass and BMD analyses were measured by DXA. The model of the equipment used was iDXA (GE Healthcare Lunar, Madison, WI, United States) and the Encore^TM^ 2011 software, version 13.6. Before players were scanned, they were warned about the use of jewelry and the presence of some types of metal in the body. For scanning, players remained in the supine position with arms extended to the side of the body and with the knees and ankles fastened by a Velcro tape. This allowed ensuring the default position. Reference points were adjusted according to the lines showed by the software. The whole-body scan was held, and the values (%) of body fat, bone mass, fat mass, FFM, and BMD were obtained.

Every day, before starting each assessment, the two evaluators in charge calibrated the equipment according to the manufacturer’s specifications. The evaluators were qualified for the professional and scientific use of the equipment according to the manufacturer’s instructions. To verify reproducibility (test and re-test), scanning was repeated on 10 soccer players. The intra-rater Technical Error of Measurement was less than 1.5%.

### Statistical Analysis

Study variables were described using descriptive statistics of the arithmetical mean (X) and the standard deviation (SD). Differences between the two groups of soccer players were verified through the *t*-test for independent samples. Relationships between variables were obtained through Pearson’s correlation coefficient. To develop the regression equations, multiple regression analysis was carried out. The equations were analyzed using *R*^2^, Standard Error of Estimation (SEE), and the Variation Inflation Factor (VIF). The Bland–Altman Plot (Bland and Altman, 1986) was used to verify the consistency between the reference values (DXA) and the equations developed. One-way ANOVA test were used to determine differences between the averages of the three predictive models. A significance level of 0.05 was adopted. Calculations were carried out with Excel worksheets and SPSS 16.0 software.

## Results

### Characteristics of Study Population

The anthropometric profile, FFM, and BMD values of the young soccer players are shown in Table 1. No significant differences occurred between both groups regarding chronological age, length of the foot, diameter of the humerus, and the BMD (*p* > 0.05). However, goalkeepers showed greater weight, stature, sitting height, arm circumference, and FFM in relation to field players (*p* < 0.05). In addition, presented the PHV first than the field players.

**Table 1:** Variables that characterize the studied sample in goalkeepers and field players.

	Goalkeepers		Field players		All	
Variables	(*n* = 22)		(*n* = 145)		(*n* = 167)	
			
	*X*	*SD*	*X*	*SD*	*X*	*SD*
Age (years)	14.7	2.4	15.2	2.4	14.9	2.4
Biological age (PHV)	13.80*	0.1	14.7	0.6	14.6	0.6
Weight (kg)	69.2*	15.1	60.5	13.1	61.7	14.1
Height (cm)	177.7*	12.9	167.2	16.5	168.7	16.6
Sitting height (cm)	90.5*	7.7	87.2	6.5	87.7	6.8
Length of the foot (cm)	26.8	1.5	25.3	1.59	25.5	1.7
Arm circumference (cm)	26.6*	3.3	24.4	3	24.8	3.2
D. Humerus (cm)	7.0	0.8	6.7	0.7	6.7	0.7
BMD (g/cm^2^)	1.2	0.2	1.2	0.2	1.2	0.2
FFM (kg)	50.0*	16.1	47.6	11.9	47.4	13.3


*X, average; SD, standard deviation; PHV, peak height velocity; C, circumference; D, diameter; BMD, bone mineral density; FFM, fat free mass; ^∗^p < 0.05.*

### The Relationship Between the Variables of Fat-Free Mass and Bone Mineral Density With Age

The linear regression analysis for the dependent variables of FFM and BMD for both groups of soccer players is described in Table 2. A positive relationship occurred between chronological age and maturity status with the FFM and BMD in both groups. A positive relationship existed between chronological age and maturity status with the FFM and BMD in both groups of soccer players. In addition, weight and arm circumference strongly related to the FFM weight, length of the foot, diameter of the humerus, and the BMD in both groups. Maturity status explained the greater % of the FFM and the BMD in relation to chronological age.

**Table 2:** Multiple linear regression values of FFM and BMD as dependent variables.

Dependent variables	Independent variables	Goalkeepers				Field players				All			
				
		*R*	*R*^2^	SEE	*p*	*R*	*R*^2^	SEE	*p*	*R*	*R*^2^	SEE	*p*
FFM (kg)													
	Chronological age (years)	0.78	0.60	1.09	0.00	0.63	0.40	1.00	0.00	0.64	0.41	1.04	0.00
	Biological age (PHV)	0.81	0.66	1.01	0.00	0.69	0.48	0.93	0.00	0.71	0.51	0.94	0.00
	Weight (kg)	0.78	0.60	1.08	0.00	0.79	0.62	0.79	0.00	0.78	0.61	0.84	0.00
	Arm circumference (cm)	0.66	0.43	1.29	0.00	0.72	0.52	0.89	0.00	0.70	0.50	0.94	0.00
BMD (g/cm^2^)													
	Chronological age (years)	0.91	0.82	0.08	0.00	0.78	0.62	0.12	0.00	0.80	0.64	0.11	0.00
	Biological age (PHV)	0.92	0.85	0.08	0.00	0.81	0.66	0.11	0.00	0.83	0.69	0.11	0.00
	Weight (kg)	0.90	0.82	0.09	0.00	0.84	0.71	0.10	0.00	0.84	0.70	0.10	0.00
	Length of the foot (cm)	0.79	0.62	0.12	0.00	0.68	0.47	0.14	0.00	0.67	0.45	0.14	0.00
	D. Humerus (cm)	0.82	0.68	0.11	0.00	0.69	0.47	0.14	0.00	0.70	0.50	0.13	0.00


*PHV, peak height velocity; C, circumference; D, diameter; BMD, bone mineral density; FFM, fat free mass; SEE, standard error of estimation*.

### Proposed Equations

The proposed equations for the FFM and the BMD for goalkeepers and field players are illustrated in Tables 3, 4. In both groups, the equations that predict the FFM are based on maturity status, weight, and arm circumference. The *R*^2^ values oscillate between 66 and 68% in goalkeepers and between 62 and 69% in field players.

**Table 3:** Regression equations to predict the FFM of goalkeepers and field players.

Groups	Prediction equations	C	VIF	*R*	*R*^2^	SEE	*p*
Goalkeepers							
1	FFM = -9462.85 + 859.08^∗^Weight	–	–	0.78	0.66	1.00	0.000
2	FFM = 25632.194 + 4315.34^∗^PHV + 321.951^∗^Weight	–	–	0.82	0.68	1.00	0.000
	PHV	0.24	4.23				
	Weight	0.24	4.23				
3	FFM = 32827.029 + 4149.134^∗^PHV + 474.071^∗^Weight-664.695^∗^AC	–	–	0.82	0.68	1.30	0.000
	PHV	0.23	4.38				
	Weight	0.10	10.00				
	Arm circumference	0.18	5.60				
Field player							
1	FFM = 2428.7 + 734.7^∗^Weight	–	–	0.81	0.66	0.10	0.000
2	FFM = 1949.902-65.081^∗^PHV + 742.903^∗^Weight	–	–	0.79	0.62	0.70	0.000
	PHV	0.22	4.61				
	Weight	0.22	4.61				
3	FFM = 4033.066-16.121^∗^PHV + 767.570^∗^Weight-146.733^∗^AC	–	–	0.83	0.69	0.10	0.000
	PHV	0.37	2.70				
	Weight	0.42	2.39				
	Arm circumference	0.40	2.48				


*C, collinearity; VIF, variation inflation factor; SEE, standard error of estimate; PHV, peak height velocity; AC, arm circumference; FFM, fat free mass*.

**Table 4:** Regression equations to predict the BMD of goalkeepers and field players.

Groups	Prediction equations	C	VIF	*R*	*R*^2^	SEE	*p*
Goalkeepers							
1	BMD = 0.802 + 0.030^∗^PHV + 0.008^∗^weight			0.92	0.85	0.08	0.000
	PHV	0.24	4.23				
	Weight	0.24	4.23				
2	BMD = 0.867 + 0.071^∗^PHV + 0.002^∗^L. Foot + 0.037^∗^D. Humerus			0.93	0.86	0.08	0.000
	PHV	0.21	4.8				
	Length of the foot (cm)	0.28	3.57				
	Diameter of the humerus	0.27	3.72				
3	DMO = 1.088 + 0.052^∗^PHV + 0.006^∗^Weight-0.016^∗^L. Foot + 0.017^∗^D. Humerus			0.95	0.90	0.07	0.000
	PHV	0.17	5.97				
	Weight	0.19	5.20				
	Length of the foot (cm)	0.25	4.02				
	Diameter of the humerus	0.26	3.89				
Field player							
1	BMD = 0.712 + 0.030^∗^PHV + 0.008^∗^Weight			0.85	0.73	0.10	0.000
	PHV	0.22	4.61				
	Weight	0.22	4.61				
2	BMD = 0.495 + 0.057^∗^PHV + 0,017^∗^L. Foot + 0.036^∗^D. Humerus			0.83	0.69	0.11	0.000
	PHV	0.37	2.7				
	Length of the foot (cm)	0.42	2.39				
	Diameter of the humerus	0.40	2.48				
3	BMD = 0.856 + 0,028^∗^PHV + 0.009^∗^Weight-0.011^∗^L. Foot + 0.010^∗^D. Humerus			0.86	0.73	0.10	0.000
	PHV	0.21	4.78				
	Weight	0.11	8.95				
	Length of the foot (cm)	0.27	3.77				
	Diameter of the humerus	0.37	2.71				


*C, collinearity; VIF, variation inflation factor; SEE, standard error of estimate; PHV, peak height velocity; L. Foot, length of the foot; D. Humerus, diameter of the humerus; BMD, bone mineral density*.

The equations that predict the BMD in both groups (goalkeepers and players) are based on maturity status, weight, length of the foot, and diameter of the humerus. Values of *R*^2^ fluctuated between 85 and 90% in goalkeepers and 83 and 86% in field players. No collinearity was observed in the FFM and BMD equations. In all cases, the values of the VIF were less than 10.0.

**FIGURE 1:**
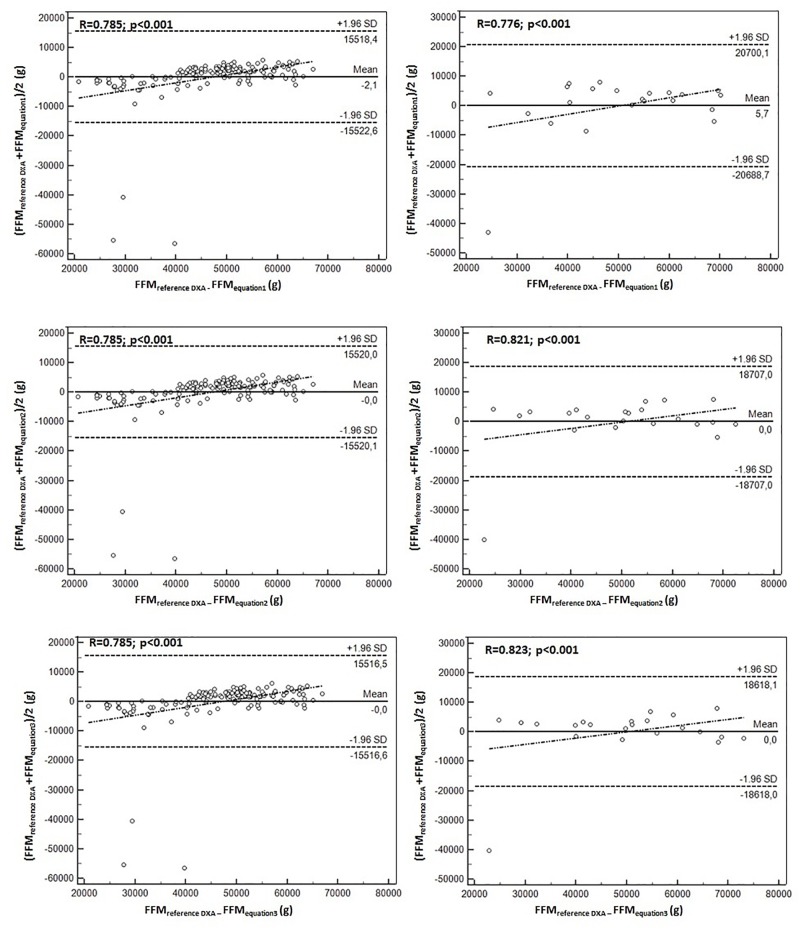
Bland–Altman plot for the correlation between the FFM values determined by the reference method (DXA) and the three predictive equations for goalkeepers (left) and field players (right).

Figure 1, 2 illustrate the correlation between the DXA method reference and the equations generated to predict the FFM and BMD in goalkeepers and field players. Overall, the 12 equations developed demonstrated broad limits of agreement in relation to the reference method. The equations generated to predict the FFM in both groups of young soccer players varied between -2.1 and 2.0. For the BMD, they oscillated between -0.38 and 0.21. Furthermore, correlations in the 12 equations were significant (*p* < 0.001).

**FIGURE 2:**
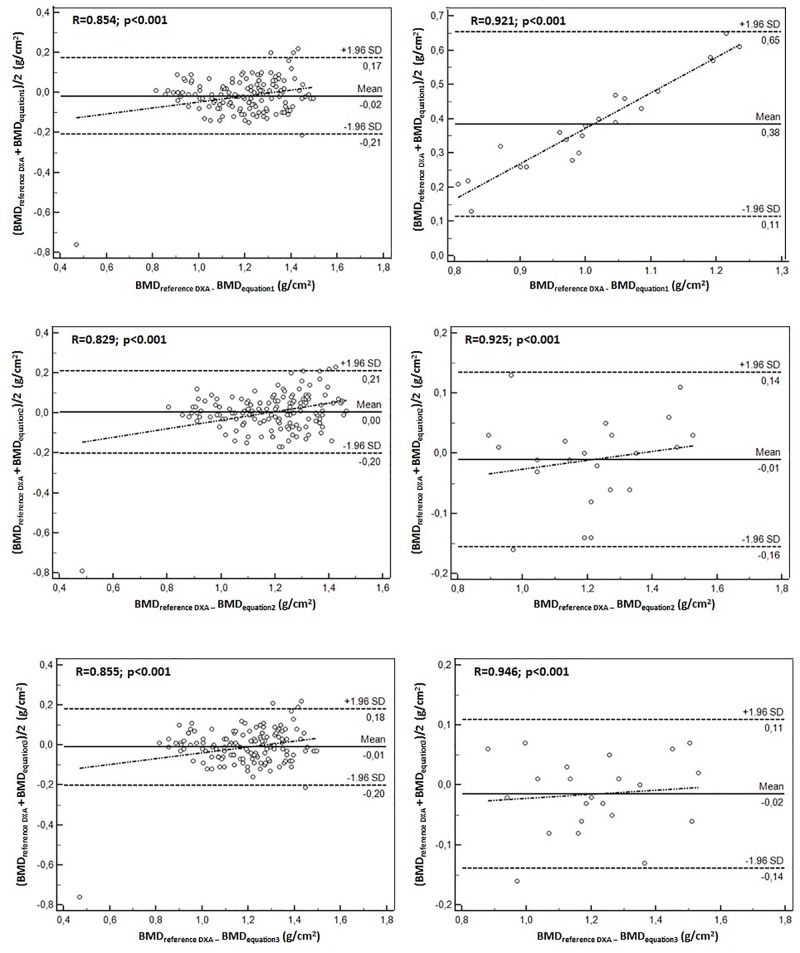
Bland–Altman plot for the correlation between the BMD values determined by the reference method (DXA) and the three predictive equations for goalkeepers (left) and field players (right).

Figure 1, 2 show the correlation between the reference method of DXA and the equations generated to predict the FFM and BMD in goalkeepers and field players. Overall, the 12 developed equations showed broad limits of agreement in relation to the reference method. The equations generated to predict the FFM in both groups oscillate between -2.1 and 2.0 and for the BMD, oscillate between -0.38 and 0.21. Furthermore, correlations in the 12 equations are significant (*p* < 0.05).

## Discussion

### Predicting FFM and BDM

The objective of this study was to determine the predictors of the FFM and BMD for young soccer players. The results from this research suggest that maturity status predicts the FFM and BMD at a higher percentage than does chronological age. This pattern was observed in the two groups: goalkeepers and field players. In fact, the status maturity plays a major role during the adolescence stage: since the chronology, duration and Intensity of the puberty are specific for each adolescent and may considerably vary among them (Malina et al., 2004).

Essentially, the results obtained from this study confirmed that chronological age is of limited use to analyze and/or study the FFM and BMD of young soccer players at least in an age group between 10.0 and 19.9 years. Therefore, the control of maturity status in young soccer players is a priority, especially to avoid potential confusion between the adolescents of various categories and age groups. Several previous studies have shown its utility and importance in samples of young soccer players (Seabra et al., 2012; Lautaro and Cossio-Bolaños, 2014; Santi-Maria et al., 2015) with grouping strategies between the soccer players helping to maintain, protect and/or promote players according to their actual state of maturation (Malina et al., 2005).

Regarding the anthropometrical variables that predict the FFM, the results demonstrated that body weight and arm circumference presented high predictive values. These results are consistent with studies carried out with adults (Lee et al., 2000; Kuriyan and Kurpad, 2004; Lyra et al., 2012) to the extent that they showed significant correlations with the FFM.

The predictors of the BMD, weight, length of the foot, and diameter of the humerus were the variables that showed high predictive values in goalkeepers and field players. These results are consistent with other studies conducted with non-athletic children and adolescents (Silva et al., 2006; Fonseca et al., 2008; Gómez-Campos et al., 2017). In addition, the results demonstrated that anthropometric variables that are routinely assessed in the field are necessary to use to analyze bone health in children and adolescents.

In this context, Henderson et al. (2002) pointed out that the BMD relates to growth and nutritional factors. This information indicates that proper nutrition may have a significant impact on body weight and a consequent increase in length and diameter of the bones (Molgaard et al., 1997). Therefore, the presence of short and narrow bones may lead to a reduction in BMD and Bone Mineral Content (BMC) and possibly to other consequences for overall health (Gómez-Campos et al., 2017).

### Equations for Goalkeepers and Field Players

The second objective of this study sought proposed regression equations to estimate the FFM and BMD with anthropometric variables. For both groups (goalkeepers and field players) in this study, 12 equations were developed (six for the FFM and six for the BMD).

Values of *R*^2^ for the FFM for the goalkeepers oscillated between 66 and 68% and between 62 and 69% in field players. For the BMD *R*^2^, values varied between 85 and 90% in goalkeepers and between 69 and 73% in field players. However, in all cases no collinearity was observed in the equations developed, and the values of the VIF were within the limits established by the literature (Slinker and Glantz, 1985).

Essentially, the 12 proposed equations showed adequate values as the Bland–Altman plot with the DXA reference method reflected narrow limits and highly significant correlation coefficients (*r* = 0.78 to 0.94). This ensured a high precision in the equations proposed for both working groups. In addition, some previous studies have reported similar *R*^2^ values and levels of agreement for samples of children and adolescents (Cameron et al., 2004; Cossio-Bolaños et al., 2017; Gómez-Campos et al., 2017).

As a result, the equations developed are a non-invasive alternative to estimate the FFM and BMD of young soccer players. These results confirm that maturity status and anthropometric variables, such as weight, arm circumference, diameter of the humerus, and the length of the foot, need be introduced into anthropometric assessment of young soccer players. These variables are simple and easy to evaluate in laboratory conditions and require tools easy to access and of low cost. These factors facilitate using the independent variables for predicting FFM and BMD of young soccer players.

The equations created are subject to bias in the calculations. As a result, precision may be compromised when used with individuals with different anthropometric characteristics, particularly with samples from different geographical locations. Even though it was not possible to use an additional group to develop cross-validation, this does not invalidate the results. Validity was ensured with the measurement of the anthropometric variables by using a standardized protocol and the DXA reference method with a sample of young players from a different professional team from Brazil.

The results from this study need to be interpreted carefully. Consequently, as far as the equations are used, external validation will be achieved.

### Limitations of the Study

Chronological age has a limited use for predicting FFM and BDM. However, the other anthropometric variables, such as weight, arm circumference, diameter of the humerus, and length of the foot, enabled generating regression equations to estimate the FFM and BDM of goalkeepers and field players.

## Conclusion

The results from this study suggest broad agreement. Furthermore, they support reproducibility of the equations. In addition, the findings demonstrated the use and application of the regression equations as a non-invasive alternative for everyday use in soccer clubs.

## Data Availability

Publicly available datasets were analyzed in this study. This data can be found here: https://figshare.com/s/fa5ff23c007cd6fa23a5.

## Author Contributions

MA, RG-C, and MC-B contributed to the conception and design of the current work, data analyses, data interpretation, and drafted the manuscript. TS-M, TM, AA, and MS coordinated NU-AGE data collection. All the authors contributed to interpretation of data, critically revised, and approved the final version of this manuscript.

## Conflict of Interest Statement

The authors declare that the research was conducted in the absence of any commercial or financial relationships that could be construed as a potential conflict of interest.
