# Supplementary material for: Fat-Free Mass and Bone Mineral Density of Young Soccer Players: Proposal of Equations Based on Anthropometric Variables
Source: Front Psychol. 2019 Mar 29;10:522. doi: 10.3389/fpsyg.2019.00522 (PMC6449479; doi:10.3389/fpsyg.2019.00522)
Supplement: Supplementary file 1 [file Data_Sheet_1.PDF]

Nro	Pos_cate	Data_Nasc	Weight	Height	L. Foot	APHV
1	Field players	15-jun-03	34,2	143,5	22,4	-3,3
2	Field players	09-feb-03	31,5	136,4	22,6	-3,5
3	Field players	22-ene-03	37,9	145	23,9	-3,1
4	Field players	29-ene-03	38,8	147	23,8	-3,1
5	Field players	30-oct-01	54,8	161,5	25,4	-2
6	Field players	10-jul-01	56,6	166,3	25,3	-1,2
7	Field players	30-may-01	43,8	150,8	22,8	-2
8	Field players	15-feb-00	53,6	166	25,1	-0,7
9	Field players	10-mar-00	54,2	159	24,7	-0,8
10	Field players	20-ene-00	53,5	166	24	-0,5
11	Field players	12-may-99	63,8	169,5	24,9	0,5
12	Field players	13-mar-99	65,9	180	27	0,8
13	Field players	04-mar-99	63,4	164,5	25,6	-0,1
14	Field players	10-mar-99	56,9	166,8	24,8	0,1
15	Field players	02-feb-99	64,7	172,5	25,4	0,1
16	Field players	04-ene-99	74,8	173,5	27	0,9
17	Field players	28-jul-98	52,6	167	24,4	0,1
18	Field players	18-jun-98	67,1	168,3	25,5	0,1
19	Field players	21-mar-98	69,8	166,5	26,1	0,5
20	Field players	07-feb-98	71,9	188	27	1,9
21	Field players	03-abr-97	70,3	174	26,2	1,1
22	Field players	15-abr-97	72,8	178,5	27	1,6
23	Field players	30-mar-97	85,1	178	26,7	2,1
24	Field players	18-jun-96	80	188	28,4	2,6
25	Field players	27-abr-96	70,1	179	27,2	2,4
26	Field players	24-mar-96	78,9	182	27	2,6
27	Field players	26-dic-95	76,7	179,5	26	2,7
28	Field players	10-abr-95	69,7	187	26,1	2,4
29	Field players	28-sept-94	63,2	167	24,5	2,4
30	Field players	21-feb-94	85	180	27,8	3,7
31	Field players	12-ene-94	64,6	172,5	25,5	3,3
32	Field players	25-sept-03	30,6	142	22	-3,4
33	Field players	14-jul-03	25,9	132	20	-3,8
34	Field players	01-abr-03	36,8	145	22,9	-3,1
35	Field players	24-mar-03	31,2	137	21,3	-3,5
36	Field players	10-feb-03	30,6	143	22,8	-3,2
37	Field players	26-jul-01	36,5	157	24,6	-2,2
38	Field players	24-ene-01	54	168	25,9	-0,8
39	Field players	16-nov-00	48,5	163,5	24,2	-1,5
40	Field players	10-ago-00	60	160	24,9	-1
41	Field players	05-abr-00	59,5	168,3	26,1	-0,6
42	Field players	12-ene-00	58,9	164,5	26	-0,8
43	Field players	23-oct-99	60,8	167,3	24,5	-0,7
44	Field players	29-sept-99	56,2	169,5	27,1	-0,8
45	Field players	24-jun-99	48,2	162,5	24,1	-0,7
46	Field players	25-mar-99	66,8	176	26,4	0,8

47	Field players	06-mar-99	60,2	175	25,9	0,5
48	Field players	11-ene-99	57,5	172,8	25,6	0,4
49	Field players	07-ene-99	69	171	24,7	0
50	Field players	29-may-98	61,3	173	27	0,7
51	Field players	11-may-98	62,4	174	26,7	0,8
52	Field players	25-feb-98	59,9	164	25	1
53	Field players	21-feb-98	70,8	176	26	1,7
54	Field players	25-nov-97	60,5	170,8	25,4	0,5
55	Field players	12-mar-97	64	171	24,6	1,3
56	Field players	04-ene-97	62,7	177	25,2	1,9
57	Field players	15-nov-96	77,1	177	27	2,1
58	Field players	18-nov-95	81,4	180,5	27,1	2,5
59	Field players	22-ago-95	66,9	171	24,6	2,2
60	Field players	17-jul-03	39,1	144	21,9	-3,1
61	Field players	30-jun-03	38,3	136,5	21,9	-3,5
62	Field players	26-feb-03	34,6	147	21,9	-2,8
63	Field players	25-sept-01	36,7	150	22,6	-2,2
64	Field players	13-jul-01	61,4	174	25,8	-0,9
65	Field players	02-jul-01	44,2	149,8	22,8	-2,4
66	Field players	09-jul-01	43,1	149	24,4	-2,3
67	Field players	21-jun-01	46,1	160	24,5	-1,9
68	Field players	20-jun-00	53	150,5	22,8	-1,4
69	Field players	26-may-00	53	160,3	23,8	-0,9
70	Field players	04-may-00	52,5	162	24,4	-1,1
71	Field players	07-may-00	44,7	154	23	-1,4
72	Field players	14-abr-00	47	153,7	23	-1,5
73	Field players	14-abr-00	49,7	168	25	-0,6
74	Field players	08-mar-00	52,6	160	23	-0,6
75	Field players	30-abr-99	63,7	170,4	25	0,3
76	Field players	01-mar-99	61	163,5	25,4	0,1
77	Field players	27-feb-99	50,5	167	25,3	0,2
78	Field players	20-jul-98	60,4	178	26	0,8
79	Field players	24-mar-98	63,2	173	26,2	0,9
80	Field players	07-may-97	69,6	175,3	25,6	1,2
81	Field players	27-mar-97	65,3	181,5	26,8	1,6
82	Field players	30-jul-96	61,7	171	24,6	1,4
83	Field players	01-feb-96	63,1	176,5	24,5	2,6
84	Field players	03-sept-95	66,3	173	24,6	1,9
85	Field players	01-jul-95	62,3	168,5	25,8	2
86	Field players	05-ago-95	64,1	172	24,9	2,7
87	Field players	20-mar-95	56,4	169	25	1,8
88	Field players	20-mar-95	56	169	24,6	1,5
89	Field players	22-ene-95	64,5	171,5	24,3	3
90	Field players	18-ene-95	62,9	173,5	25,6	2,9
91	Field players	05-ago-94	76,9	182	26,4	3,3
92	Field players	06-feb-03	46,2	142	22	-3,1
93	Field players	24-ene-03	38,9	144,3	23,5	-3

94	Field players	16-oct-01	33,5	142	21,9	-2,3
95	Field players	23-may-01	51,8	156	25,7	-1,9
96	Field players	29-may-01	39,2	149	23	-2,2
97	Field players	07-mar-01	52,2	166	24,7	-1,4
98	Field players	24-ene-01	52,5	165	25,4	-1
99	Field players	07-sept-00	50,8	162,5	23,7	-1,1
100	Field players	22-jul-00	56,1	162	25,4	-1,1
101	Field players	10-mar-00	60,4	179	24,9	0,4
102	Field players	01-mar-00	55,5	171	26,9	-0,4
103	Field players	20-ene-00	51,6	163,6	25,1	-0,2
104	Field players	19-ene-00	54,3	170,5	23,9	0,1
105	Field players	19-dic-99	63,7	176,5	25,4	0,4
106	Field players	13-may-99	67,5	175,8	27,4	0,2
107	Field players	13-abr-99	65,2	168	24,7	0,3
108	Field players	21-feb-99	63,1	168,5	25	-0,3
109	Field players	23-sept-98	70,6	178	27,1	0,9
110	Field players	24-ago-98	72,4	175,5	25,7	0,5
111	Field players	18-abr-98	61,2	176	24,4	1,8
112	Field players	21-feb-98	77,5	178,5	27,1	1
113	Field players	27-feb-98	68,1	178,5	26	1,5
114	Field players	28-dic-97	71,3	166,5	25,5	1
115	Field players	20-mar-97	69,5	165	26	0,8
116	Field players	07-ene-97	70,3	173	26	1,7
117	Field players	13-may-96	61,5	170	25	1,8
118	Field players	13-may-95	69,4	74,5	26,3	3,1
119	Field players	13-may-95	75,3	187	27,5	3,5
120	Field players	05-ago-94	79,2	181,5	26,3	3,1
121	Field players	25-may-94	82,5	176	25,6	3,7
122	Field players	09-jun-94	86	185	26,5	3,6
123	Field players	19-ene-94	89,2	188,5	28	4,3
124	Field players	06-ago-01	59,9	170,5	25,6	-1
125	Field players	26-abr-01	60,9	161,5	25,5	-1,4
126	Field players	30-ene-01	43,9	154	23,6	-1,8
127	Field players	01-ago-00	62,9	174,5	25,6	-0,6
128	Field players	31-mar-00	54,9	182,2	26	0,2
129	Field players	17-mar-00	57,1	169,5	24,4	-0,2
130	Field players	29-ago-99	72,5	187	27	1
131	Field players	01-mar-99	64,1	176	27,4	0,6
132	Field players	10-ene-99	80,9	186,5	28	0,8
133	Field players	01-ene-99	66,5	181	26,6	1
134	Field players	23-sept-98	75,7	185	27,5	1,4
135	Field players	12-jun-98	75,5	184	28	0,9
136	Field players	19-feb-98	76,3	182	27,2	1,4
137	Field players	14-jul-97	83,2	185	28,8	2,2
138	Field players	12-jun-97	73,5	175	26,8	1,5
139	Field players	16-abr-97	78,1	185	28,5	2,1
140	Field players	12-ene-97	72,7	183,5	27,7	2,3

141	Field players	05-ene-97	79,4	83,5	28,1	2,7
142	Field players	27-oct-96	64,1	187	26,6	2,2
143	Field players	25-feb-96	75,9	195,3	28,2	3,1
144	Field players	05-nov-94	79,4	185,5	27,2	4,2
145	Field players	10-may-94	76	180,5	25	3,7
146	Goalkeepers	07-oct-03	57	158,5	25	-3,2
147	Goalkeepers	25-may-03	49,8	153	23,6	-3
148	Goalkeepers	24-may-03	37,1	146	23,5	-3,5
149	Goalkeepers	11-jun-01	57,3	166,5	25,8	-1,4
150	Goalkeepers	12-mar-01	53,5	173,2	26,4	-0,9
151	Goalkeepers	22-feb-01	66,7	167	26,3	-1,2
152	Goalkeepers	16-sept-00	64,2	173	26,1	-0,8
153	Goalkeepers	24-abr-00	72,1	176,5	27,2	0,2
154	Goalkeepers	18-mar-00	73,8	187	28,2	0,4
155	Goalkeepers	08-feb-00	53,4	171,5	25,3	-0,1
156	Goalkeepers	15-sept-99	65,7	182	27,5	0,5
157	Goalkeepers	23-abr-99	78,2	185,5	28	0,9
158	Goalkeepers	03-mar-99	59,9	185,2	27,1	1,1
159	Goalkeepers	08-mar-99	73,3	184,5	27,2	0,6
160	Goalkeepers	03-may-98	60,2	176,3	26,5	1,2
161	Goalkeepers	11-abr-98	74,1	187	28	1,6
162	Goalkeepers	05-mar-98	80,5	191	28,7	2,1
163	Goalkeepers	04-jul-97	90,5	186,5	28,1	2,2
164	Goalkeepers	05-ago-96	91,4	188	28,2	3
165	Goalkeepers	31-ene-96	81,6	186,5	27,7	3,1
166	Goalkeepers	04-sept-95	94,1	191	27	3,6
167	Goalkeepers	28-mar-94	89	194	28,3	4,3

Arm circunferen	D. Humerus	BMD	FFM
19	8,6	0,89	25714
18,9	8	0,95	24289
18,4	8,6	1,01	28962
19,9	8,6	0,93	28336
22,4	9,4	1,11	44156
23	9,2	1,13	43864
21,7	8,7	1,01	32582
22,7	9,6	1,17	43678
23,2	9,6	1,06	38102
22	9,5	1,05	45010
25,6	9,8	1,19	51862
24,1	9,8	1,18	54192
25,3	9,5	1,23	51228
24,7	9,4	1,24	45611
23,9	9,2	1,29	51801
26	10,7	1,25	57503
23,8	8,2	1,24	44285
25,5	9,8	1,14	53494
28	10,3	1,25	54483
26,3	10,1	1,25	54471
28,5	9,6	1,47	60002
27,4	9,6	1,25	59095
30,6	10,7	1,43	65222
28,2	10,4	1,47	66560
27,2	8,6	1,22	55721
28,3	10,1	1,34	64403
28,9	9,5	1,31	58960
27	9,2	1,46	58378
26,5	9,5	1,37	52275
32,2	9,5	1,47	62266
26,8	9,6	1,25	9165
18,7	7,9	0,09	23973
17,1	7,3	0,82	19962
19,1	8,6	0,91	26096
17,3	8,4	0,85	23431
18,2	7,7	0,87	23834
18	8,2	0,99	29054
21,5	9,6	1,17	45536
20,2	8,9	1,05	37395
24,3	9,4	1,11	42887
22,5	9,7	1,15	49685
24,2	9,8	1,15	47821
26,4	9,8	1,28	49187
22	9,1	1,23	44125
20,8	9,4	1,01	37943
24,2	9,8	1,28	53331

22,7	9	1,09	50366
22,9	9,6	1,16	46818
24,3	9,4	1,36	52115
24,9	9,8	1,13	48935
25,1	10,2	1,13	51909
25,2	9,3	1,31	47848
27	9,9	1,3	54034
24,8	9,2	1,41	51784
26,8	9,3	1,3	52000
24	9,5	1,21	51409
30,8	9,8	1,28	63087
31,4	10,1	1,33	64293
27,5	9,1	1,51	53272
20,6	8,4	0,91	27910
21,6	8,8	0,97	26175
18,4	8,3	0,97	25803
18,3	8,5	1	26238
23,7	9,8	1,11	48888
21,8	9,1	1,02	30471
21,6	9,2	0,98	31973
20,2	8,8	0,99	35661
24	9,1	1,04	39281
23,3	8,2	1,18	42767
22,5	8,6	1,09	41852
22	9	1	35479
23,7	9,2	0,96	34057
21,5	9,1	1,05	38951
23,2	9	1,13	42700
25,7	9,8	1,29	51516
25,5	9,8	1,22	49419
22,2	9,1	1,04	42085
23,5	9,3	1,14	49639
25,6	9,2	1,19	53982
26	10,2	1,29	55389
26,6	9,5	1,21	50622
25	9,1	1,27	49567
25,9	9,2	1,14	51403
26,4	9,8	1,31	53917
27,5	9,6	1,13	52257
26,4	9,4	1,21	52015
25,9	8,8	1,32	47742
26,2	8,6	1,3	46425
27	8,5	1,54	52630
25,8	9,4	1,39	53092
26,7	9,3	1,34	58912
23,3	8,5	0,92	27233
20,8	8,5	0,89	27145

19,5	8,1	0,91	26428
22,7	9,5	1,04	33666
20	8,6	0,95	31481
22	8,8	0,96	40301
21,2	9,6	1,12	43556
21,3	9,8	1,12	41034
24,3	9,2	1,08	40779
24	9,3	1,18	49601
22,3	10,1	1,12	45330
22,7	8,8	0,98	42105
22,4	9	1,2	45236
25	9,4	1,27	51550
26,2	9,8	1,27	56417
24,7	10,2	1,21	53334
24,9	9,5	1,24	49851
26	9,9	1,34	56426
27,8	9,6	1,38	53295
23,5	10,1	1,2	51415
28,4	9,8	1,34	63038
26,9	9,5	1,36	54561
27,1	9,5	1,19	53428
27,1	9,5	1,35	51224
26,9	8,8	1,23	56635
24,1	9,2	1,27	50647
27,4	9,5	1,38	56688
27,7	9,5	1,32	62621
27,1	9,7	1,35	61324
29,9	10,1	1,47	63885
30,2	9,5	1,48	68293
30,2	9,7	1,34	11477
25,1	9,6	1,1	49406
23,2	9,9	1,04	45046
22,1	8,5	1,08	30344
25,5	9,7	1,18	51805
21,6	9,6	1,02	43897
22,3	8,8	1,11	47029
24,9	10,6	1,35	57956
23,4	9,4	1,24	49457
26,5	10,5	1,45	61378
23,3	9,5	1,26	53265
27,5	9,8	1,36	60336
26	9,9	1,4	61344
27,1	10,1	1,23	58674
27,2	10,7	1,37	62707
28,1	9,8	1,36	58039
27	9,9	1,35	64985
27	9	1,35	59359

26,3	9,6	1,44	63568
24,9	9,2	1,18	53306
24,9	10,4	1,34	58860
28,7	9,9	1,45	65244
28,7	9,8	1,41	60397
23,5	8,8	1,03	33630
24,7	8,9	0,93	30811
19,8	8,6	0,91	26786
23,4	9,2	0,89	41026
22,2	9,8	1,04	43064
28,3	9,7	1,03	39210
25,5	9,4	1,14	2726,6
28,1	10,1	1,19	52766
27,6	10,3	1,28	58120
24,3	9,1	1,14	44035
24,6	9,8	1,17	52244
26,2	10,5	1,29	62126
23	9,5	1,14	47814
27,1	10	1,22	55890
25,8	9,5	1,12	50392
27,8	9,5	1,24	55848
29,2	9,8	1,35	61604
31,8	10,7	1,3	71863
28,8	10,5	1,54	67869
30,2	9,7	1,48	64425
34	9,8	1,48	66212
28,2	9,1	1,54	72081
